# Vine Tea Extract Enhanced the Fermentation of Skimmed Milk by *Lacticaseibacillus casei*

**DOI:** 10.1002/fsn3.4547

**Published:** 2024-10-22

**Authors:** Kun Wang, Chengjie Ma, Man Zhang

**Affiliations:** ^1^ State Key Laboratory of Dairy Biotechnology, Shanghai Engineering Research Center of Dairy Biotechnology Dairy Research Institute of Bright Dairy & Food Co., Ltd. Shanghai China; ^2^ Wuhan Sunma Biotechnology Corp. Donghu New & High Technology Development Zone Wuhan China

**Keywords:** antioxidant capacity, cell viability, dihydromyricetin, *Lacticaseibacillus casei*, vine tea

## Abstract

Vine tea extract (VTE), from the traditional Chinese herbal tea, was added to reconstituted skimmed milk; the mixture was fermented with *Lacticaseibacillus casei*, and fermentation characteristics, flavonoid content, antioxidant capacity (AOC), and viability of *L. casei* were measured. 2 mg/mL VTE promoted *L. casei* growth and 8 mg/mL VTE inhibited growth, an effect consistent with observed pH changes. Total flavonoid content and AOC increased with increasing VTE dosage. Dihydromyricetin was partially metabolized during fermentation and accounted for most of the antioxidant function of VTE. 2 mg/mL VTE was optimal for maintenance of probiotic culture and pH stability during cold storage and improved AOC during product shelf life. VTE has the potential to increase the health benefits of probiotic dairy products, and the resulting mixture may be suitable to use as a daily milk‐based health drink.

## Graphical abstract

Vine tea was first added to skimmed milk fermented with *Lacticaseibacillus casei*. 2 mg/mL vine tea extract could improve the fermentation characters and functionality of dairy products. Addition of VTE maintained highly viable *L. casei* and stable pH during storage.

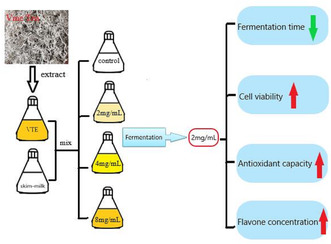

## 1 Introduction

*Nekemias grossedentata* (Hand.‐Mazz.) J. Wen & Z.L. Nie (Synonym: *Ampelopsis grossedentata* (Hand.‐Mazz.) W. T. Wang) (Haolin Zhang et al. 2024), commonly known as the Chinese vine tea plant, grows in Southwest China. The tender stem and leaves have been used for centuries as a herbal medicine by a minority group in China, and its properties have more recently been the subject of systematic study. Dihydromyricetin (DMY, ≤ 39.4% dry weight) and myricetin are the principal flavonoid components of vine tea (Luo et al. 2023; Wang et al. 2016).

DMY was shown to ameliorate non‐alcoholic fatty liver disease (NAFLD) by balancing fatty acid oxidation and lipogenesis, remitting hepatic oxidative stress, and adjusting the gut microbiome (Xie et al. 2020). DMY also had antibacterial activity against foodborne *Staphylococcus aureus*, *Bacillus subtilis*, *Escherichia coli*, *Salmonella paratyphi*, and *Pseudomonas aeruginosa* (Xie et al. 2019) in addition to anti‐inflammatory, antioxidative, hepatoprotective and anti‐carcinogenic pharmacological effects (Luo et al. 2023; Miao et al. 2023; Wang et al. 2022; Xie et al. 2019). Recent studies of mouse models of Alzheimer's disease have shown that DMY treatment reduced behavioral deficits and reversed progressive neuropathology (Liang et al. 2014). This compound also had a neuroprotective effect and restored sleep deprivation‐induced memory impairment (Li et al. 2019).

*Lacticaseibacillus casei* is used in probiotic fermented milk products, such as yoghurt. The bacteria colonize the intestine and have anti‐oxidant and anti‐inflammatory actions, protecting intestinal health and regulating the immune system (Wen et al. 2020). *L. casei* was also found to have a protective effect against prostate cancer and reduced the side effects associated with chemotherapy (Méndez Utz et al. 2020; Rosa et al. 2020). Like DMY, *L. casei* was also beneficial in treating NAFLD, reducing oxidative stress and lipid accumulation (Azarang et al. 2020; Huang et al. 2020).

*A. grossedentata* and *L. casei* appear to have complementary health properties, and vine tea extracts (VTE) have been used to increase the natural antioxidant properties of meat products, such as cooked pork patties and minced beef (Ye et al. 2015; Zhang et al. 2019). However, the effects of VTE on the probiotic fermentation of milk with *L. casei* have not been studied.

VTE was added to reconstituted skimmed milk (RSM) and fermented by *L. casei* LC2W during the current work. Fermentation characteristics, flavonoid content, antioxidant capacity (AOC), and viability of *L. casei* were monitored to determine the effects of the VTE. The aim was to investigate the possibility of using VTE in dairy product processing.

## 2 Materials and Methods

### 2.1 Preparation of VTE

Vine tea (5 g) was purchased from Zhangjiajie Qing'an Tea Co., Ltd. (Zhangjiajie, China), added to 250 mL of pure water (> 10 MΩ.cm), and boiled for 20 min. The filtrate was collected and the residue boiled again for 20 min with 125 mL of pure water. The combined filtrate was reduced to 100 mL under vacuum, refrigerated for 24 h at 4 °C, and the solid isolated using a vacuum nutsche filter. About 1.35 g crude extract was recovered and dried for 8 h at 50 °C.

### 2.2 Preparation of *L. casei* LC2W

*L. casei* LC2W was obtained from Bright Dairy & Food Co., Ltd. (Shanghai, China) and cultured on MRS agar (Merck KGaA, Darmstadt, Germany) under anaerobic conditions (95% N_2_, 5% CO_2_, 37 °C) using a Bugbox anaerobic workstation (Ruskinn Technology Ltd., Bridgend, UK). Pure single *L. casei* colonies were isolated by streak‐plate isolation and cultured to the logarithmic phase in MRS broth, centrifuged at 10,000 *g* for 10 min, rinsed twice with sterile saline solution (0.85%, w/w), and the concentration adjusted to 1× 10^9^ CFU/mL with sterile saline solution. The inoculation solution was stored at 4 °C.

### 2.3 Preparation of Milk Fermentation Samples

Skimmed milk powder (33.4% protein and 0.8% fat; Fonterra Ltd., Auckland, New Zealand) was reconstituted in pure water at 12% (w/w). Four experimental groupings were generated: Control: reconstituted skimmed milk (RSM) with no additive; RSM with 2 mg/mL VTE; RSM with 4 mg/mL VTE; and RSM with 8 mg/mL VTE. The samples were blended using an RW 20 stirrer (IKA, Staufen, Germany), sterilized at 110 °C for 5 min, and cooled to 37 °C. pH was monitored for all conditions and inoculated with *L. casei* LC2W 1 × 10^7^ CFU/mL for incubation at 37 °C for 12, 24, 36, and 48 h followed by storage at 4 °C for 28 days.

### 2.4 Measurement of pH

pH was measured at 12, 24, 36, and 48 h using a portable pH meter (Mettler‐Toledo GmbH, Greifensee, Switzerland).

### 2.5 Enumeration of *L. casei*

*L. casei* numbers were counted by diluting samples with sterile saline 10^8^ times by serial dilution. Diluted samples were cultured as above on MRS agar plates for 48 h and a colony count of 30–300 considered valid.

### 2.6 Measurement of Total Flavonoid Content

Total flavonoids were determined by aluminum chloride colorimetry using DMY as the standard.

#### 2.6.1 Preparation of DMY Standard Curve

DMY (> 98%; Anpu, Shanghai, China) was diluted to 0.2 mg/mL with 50% (v/v) ethanol and 0, 1, 2, 3, 4, and 5 mL aliquots added to 25 mL colorimetric tubes. 2.0 mL of 2.5 mg/mL AlCl_3_ and 2.0 mL of 9.82 g/100 mL potassium acetate were added, and the volume was made up to 25 mL with 50% ethanol and mixed thoroughly. The absorbance of 1 mL solution was measured at 300 nm using a UV6x Series UV spectrophotometer (Bluwave, Shanghai, China) and a standard curve plotted with DMY concentration on the abscissa and absorbance values on the ordinate.

#### 2.6.2 Analysis of Samples

Absorbances of 0.1 mL aliquots of fermented sample solutions were measured with AlCl_3_, potassium acetate, and ethanol (as above) and centrifuged for 5 min at 1500 *g* after 10 min reaction time.

### 2.7 Measurement of Antioxidant Capacity by 2,2‐Diphenyl‐1‐Picrylhydrazyl (DPPH) Assay

AOC was determined by the DPPH assay according to the method of Wang et al. (2019) with slight modifications. Briefly, a 0.1 mL sample was added to a 3.9 mL DPPH solution (0.004 g DPPH dissolved in 100 mL 95% ethanol) and incubated for 25 min at room temperature (26 °C) in the dark. The mixture was centrifuged at 1500 *g* for 5 min and absorbance measured at 517 nm with a UV6x Series UV spectrophotometer (Bluwave, Shanghai, China) with ethanol as blank. Inhibition was calculated according to Equation (1):

(1) $$\text{Percentage Inhibition}\;\left(\%\right)=\left[1-\left(\frac{A\text{sample}}{A\text{control}}\right)\right]\times 100$$

where *A*_sample_ and *A*_control_ represent the absorbance of a 0.1 mL sample and a 0.1 mL solvent, respectively. DPPH scavenging activities were determined as 6‐hydroxy‐2,5,7,8‐tetramethylchroman‐2‐carboxylic acid (Trolox) equivalents (TE) using concentrations of 50, 100, 200, and 400 mg/L to prepare a standard curve. AOC was expressed as μmol TE/g sample, which achieved 50% inhibition.

### 2.8 Measurement of DMY Concentrations in VTE and Fermented Samples

DMY standard (> 98%; Anpu, Shanghai, China) was serially diluted with methanol to give calibration standards of 5, 10, 50, and 100 μg/mL. Crude VTE (0.1 g) was dissolved in 50 mL of pure water and diluted to 10, 50, 100, and 200 μg/mL. VTE extracts and fermentation samples prepared in 2.3 were passed through a 0.22 μm filter (Millipore, USA) before analysis by HPLC (Agilent 1260 Infinity II; Agilent Technology Co., Ltd) with UV detection (G7114A 1260VWD; Agilent Technology Co., Ltd). Samples were separated on a ZORBAX‐SB C18 column (Agilent, USA; 4.6 mm × 250 mm, 5 μm) using isocratic elution with the following conditions: mobile phase: methanol/0.05% aqueous phosphoric acid solution (35:65); detection wavelength, 289 nm; flow rate, 1.0 mL/min; column temperature, 30 °C.

DMY was quantified from a linear least squares fit to the calibration data set obtained by plotting the peak area integral value as the abscissa (x) and the concentration of DMY (μg/mL) as the ordinate (*y*) (*y* = 20,429*x*—29,794, *R* ^2^ = 0.998).

### 2.9 Sample Quality During Storage

Samples were fermented for 24 h and refrigerated for 28 days, and pH, *L. casei* cell counts, and AOC were measured every 7 days.

### 2.10 Statistical Analysis

All results are expressed as mean or mean ± SD. Differences between means were assessed by one‐way ANOVA and Duncan's multiple range test (*p* < 0.05) using IBM SPSS Statistics for Windows, Version 20 (IBM Corp., Armonk, NY., USA).

## 3 Results and Discussion

### 3.1 Effect of VTE on pH of *L. casei*‐Fermented Milk

**FIGURE 1:**
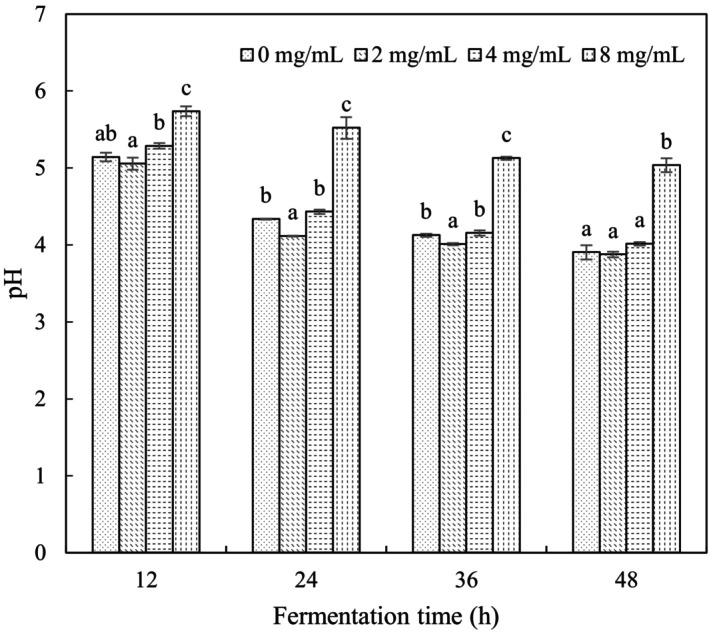
The pH changes of different VTE concentration samples. The lowercase letters indicate significant differences (*p* < 0.05) between different VTE concentrations at the same fermentation time.

pH decreased gradually with increasing fermentation time for all samples (Figure 1). 8 mg/mL VTE produced the highest pH at each fermentation time, and no coagulation was recorded. Samples with 0–4 mg/mL VTE showed the greatest pH changes over 12–24 h, and these samples all coagulated. pH reduced slowly after 24 h. 2 mg/mL VTE produced significantly lower pH at 24 h than any other condition (*p* < 0.05).

Casein has an isoelectric point of pH 4.6, below which coagulation occurs and yoghurt forms (Nogueira et al. 2019). pH changes are seen when lactic acid is produced due to the proliferation of bacteria (Prieto‐Santiago et al. 2024). We speculated that changes in pH were seen during the current experiments due to the effects of VTE in promoting or inhibiting *L. casei* growth. High VTE concentrations appeared to inhibit the acidification produced by *L. casei*, whereas 2 mg/mL VTE promoted bacterial growth, acidification, and RSM coagulation.

### 3.2 Effect of VTE on *L. casei* Viability

**FIGURE 2:**
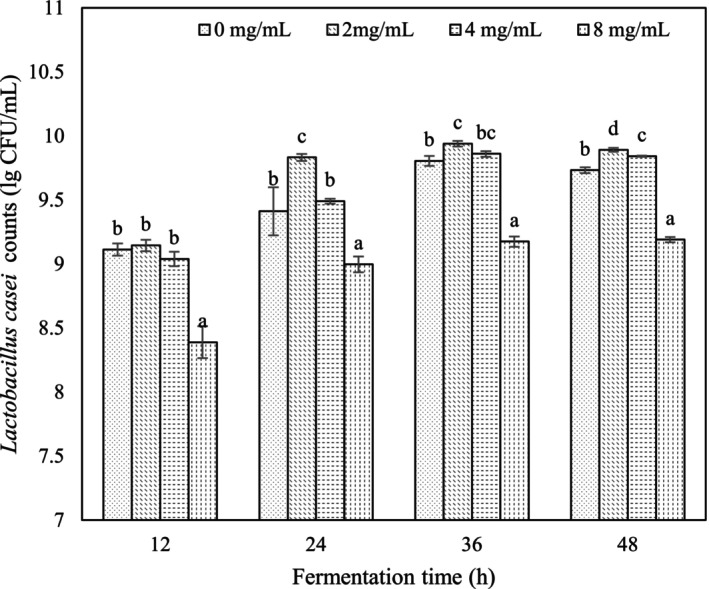
The viability changes of different VTE concentration samples. The lowercase letters indicate significant differences (*p* < 0.05) between different VTE concentrations at the same fermentation time.

*L. casei* cell counts were highest in the presence of 2 mg/mL VTE throughout fermentation and were significantly higher than control samples (*p* < 0.05) from 24 to 48 h, reaching a maximum value at 36 h and decreasing slightly thereafter (Figure 2). The addition of 8 mg/mL VTE produced significantly lower *L. casei* counts during fermentation compared with 0–4 mg/mL. Viability measurements were consistent with pH changes (Figure 1). The data support the view that 2 mg/mL VTE stimulated *L. casei* growth during RSM fermentation.

The current results show that *L. casei* did not tolerate high concentrations of VTE of 8 mg/mL. It has previously been shown that Pu‐erh tea extract promoted acidification by *Lactobacillus acidophilus* NCFM and *Lacticaseibacillus rhamnosus* GG and that high concentrations of 6% or 9% had growth‐promoting effects (Li et al. 2016). Previous studies have shown that different kinds of tea extracts had effects that were individual to the strain of *Lactobacillus*, perhaps due to differing tea ingredients. The DMY present in vine tea is known to have antibacterial activity against *Escherichia coli*, *Salmonella paratyphi*, and *Staphylococcus aureus* and may cause damage to cell walls and membranes, allowing leakage of intracellular contents with an impact on respiration (Xiao et al. 2019). The current work supports the view that the high content of DMY at higher concentrations of VTE inhibited the growth of *L. casei*.

### 3.3 AOC of *L. casei*‐Fermented Milk

**FIGURE 3:**
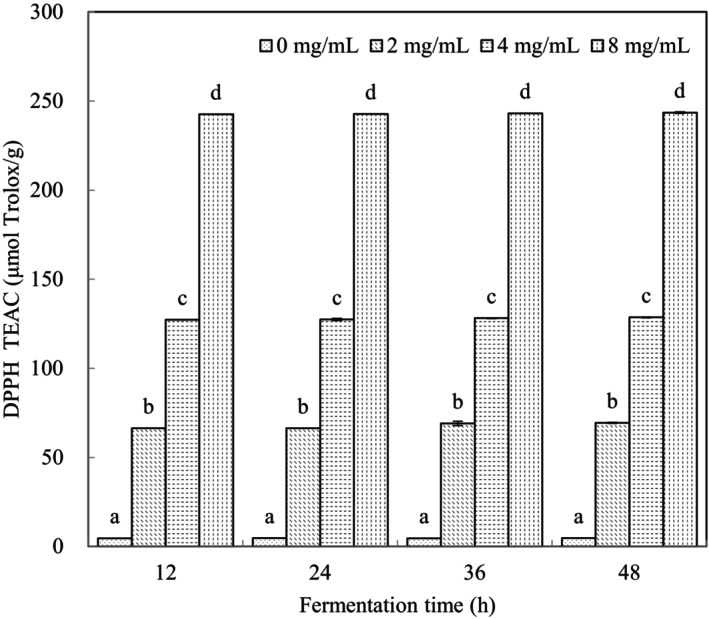
Antioxidant capacity of different VTE concentration samples. The lowercase letters indicate significant different (*p* < 0.05) between different VTE concentrations at the same fermentation time.

Dose‐dependent increases of AOC (*p* < 0.05) were seen with VTE at all time points (Figure 3). AOC increased by ~1.9 times for each doubling of VTE concentration. In addition, AOC increased with fermentation time at each VTE concentration. VTE has previously been shown to be abundant in flavonoids, of which the principal component is DMY, and these have pronounced DPPH radical scavenging activity (Xie et al. 2019; Zhang et al. 2019). Vine tea contains additional bioactive compounds, such as quercetin, kaempferol, hesperidin, and rutin, which all have anti‐oxidant and anti‐inflammatory activities. (Zhang et al. 2021). Thus, VTE components conferred the antioxidant activity on fermentation products. The increase in AOC with fermentation time at each VTE concentration may be attributed to increased *L. casei* counts. The proteolysis of milk proteins by *L. casei* led to the release of antioxidant peptides resulting in greater AOC at 36 and 48 h than at 12 and 24 h (Fardet and Rock 2017).

Lactic acid bacteria (LAB) accumulate oxidation products during proliferation, such as reactive oxygen species and hydrogen peroxide, which have a cytotoxic effect (Virolle et al. 2016; Wang et al. 2019; Zotta, Parente, and Ricciardi 2017). Green tea extract was shown to contain high levels of antioxidants with oxygen‐scavenging properties, which enhanced probiotic stability (Shah et al. 2010). VTE at 2 mg/mL increased *L. casei* viability during the current work, consistent with a favorable environment for probiotic bacterial growth.

A satisfactory level of coagulation after 24 h was found under all conditions tested in the current experiments, and AOC did not show a dramatic change with the extension of fermentation time. Consequently, 24 h was selected for subsequent experiments.

### 3.4 Total Flavonoid Content of *L. casei*‐Fermented Milks

**FIGURE 4:**
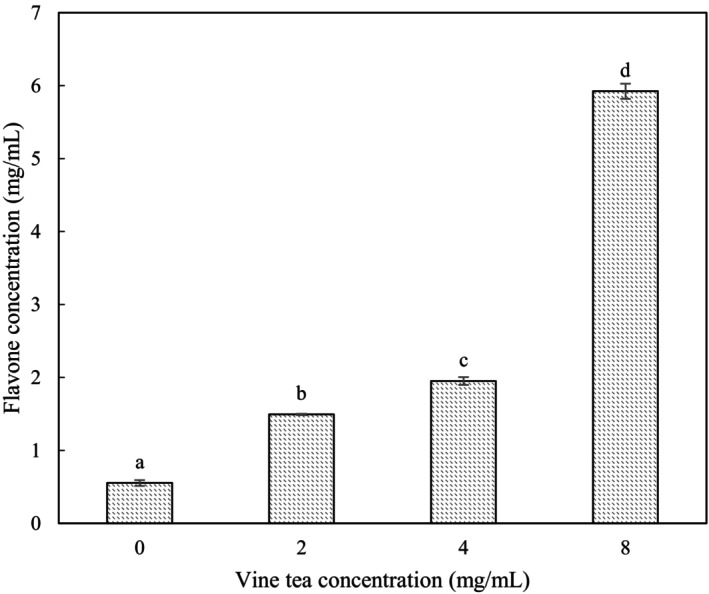
Flavone concentration of different VTE concentration samples (24 h samples). The lowercase letters indicate significant differences (*p* < 0.05) between different VTE concentrations at 24 h.

Total flavonoid content increased significantly, but in a non‐dose‐dependent manner, with each incremental increase in VTE concentration (Figure 4). These results may indicate that some flavonoid degradation took place due to bacterial proliferation (Wu et al. 2016). Decreased polyphenols at anaphase during vine tea fermentation by the fungus *Poria cocos* have been reported (Wu et al. 2016). Moreover, some lactobacilli species, such as *Lactiplantibacillus plantarum* and some gut bacteria, have been found to metabolize phenolic compounds (Loo et al. 2020; Rodríguez et al. 2009). *L. plantarum*, *Lactobacillus delbrueckii* subsp. *Bulgaricus*, and *Lactobacillus acidophilus* have rhamnosidase activity and hydrolyzed hesperidin and rutin (Mueller et al. 2017). The current colony counts of *L. casei* were lowest in samples with the highest VTE concentrations and flavonoid content. Hence, bacterial uptake of nutrient flavonoids was much lower in the RSM medium containing a higher VTE concentration and *vice versa*.

### 3.5 DMY Concentration in Vine Tea Extracts and Fermented Samples

Solutions of 50 and 100 μg/mL VTE had respective DMY concentrations of 26.1 and 51.2 μg/mL (Supporting Information). Hence, 2 mg/mL VTE is likely to be equivalent to 1 mg/mL DMY, sufficient to cause a significant increase in *L. casei* colony counts during RSM fermentation. Zhang extracted 86.46 mg DMY from 200 mg extract using methanol, giving an extraction rate of about 43.23% (Zhang et al. 2016). The current extraction method was relatively efficient by comparison.

DMY increased in fermented samples in proportion to the addition of VTE (Table 1). These results were consistent with AOC changes (Figure 3), indicating the major antioxidant role of DMY. Lower levels of DMY with lower VTE addition demonstrate the metabolism of DMY during fermentation, consistent with total flavonoid results (Figure 4). Reduced viability of *L. casei* after addition of 8 mg/mL VTE compared with 2 mg/mL illustrates the antibacterial activity of increasing DMY concentration. 0.3125 mg/mL DMY was shown to inhibit growth of *Escherichia coli* and *Pseudomonas aeruginosa* with a minimal bactericidal concentration of 2.5 mg/mL (Xiao et al. 2019). However, *L. casei* is known to have a great resistance to oxidative stress (Hosseini Nezhad, Hussain, and Britz 2014), which may account for why 3.26 mg/mL DMY was not completely cytotoxic.

**TABLE 1:** DMY concentration of 24‐h‐fermented samples.

VTE concentration (mg/mL)	Volume of equivalent DMY addition (mg/mL)	DMY concentration of fermented samples (mg/mL)
0	0	0.00
2	1	0.67 ± 0.02
4	2	1.62 ± 0.06
8	4	3.26 ± 0.09

### 3.6 Characteristics of *L. casei*‐Fermented Milk During Cold Storage

**FIGURE 5:**
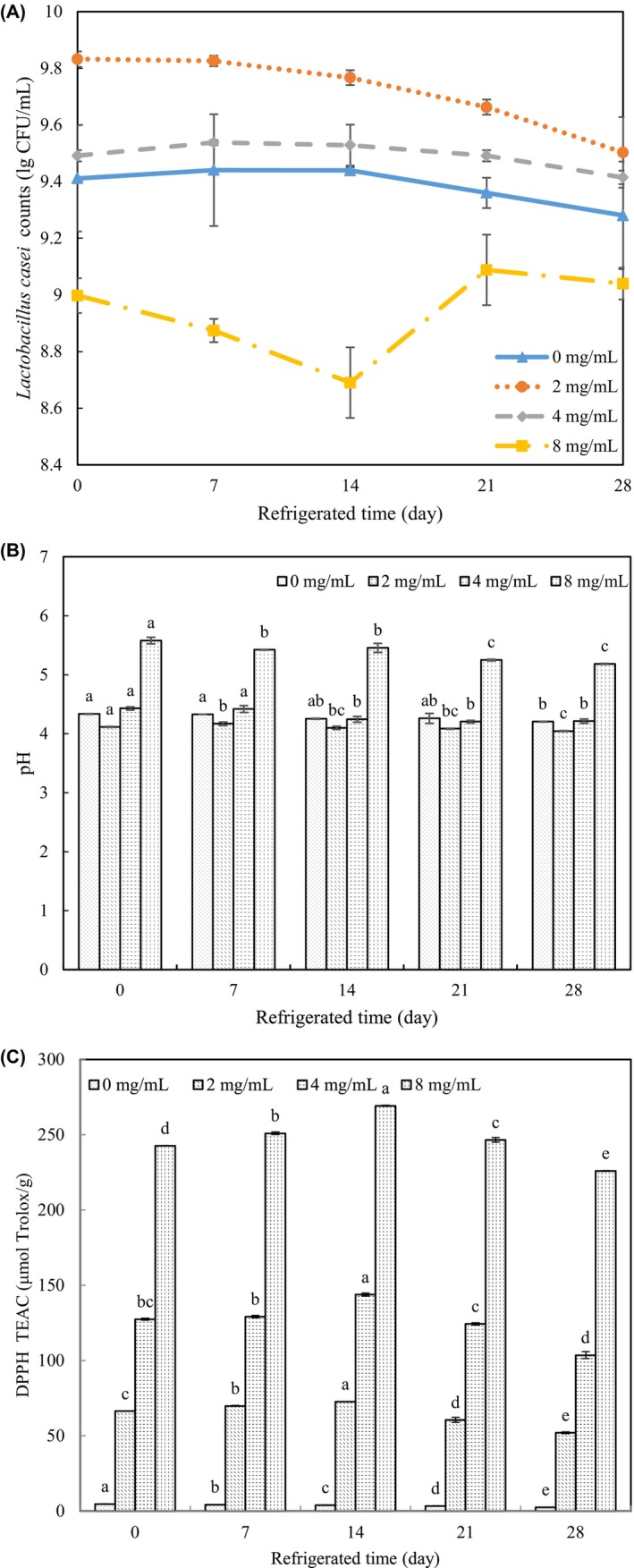
Fermentation characters of 24‐h‐fermented samples during cold storage. (A) The pH changes of 24‐h‐fermented samples during cold storage; (B) the viability changes of 24‐h‐fermented samples during cold storage; (C) the AOC changes of 24‐h‐fermented samples during cold storage. The lowercase letters indicate significant differences (*p* < 0.05) between different refrigerated times of the same VTE concentration sample.

Samples were fermented for 24 h and refrigerated for 28 days before measurement of pH, *L. casei* viability, and AOC. The highest colony numbers were seen under conditions of 2 mg/mL VTE, and these decreased gradually during storage (Figure 5a). Control counts decreased to 2 × 10^9^ CFU/mL at 28 days, and the presence of 4 mg/mL also led to a progressive decrease with storage. Colony numbers were lower than with 2 mg/mL VTE for all other conditions, and 8 mg/mL VTE showed the lowest colony counts of all, reaching a minimum at 14 days and increasing slightly thereafter. This later small increase may be due to the tolerance of some *L. casei* colonies to high concentrations of VTE during storage. A small and progressive decrease in pH at all VTE concentrations was seen during storage (Figure 5b). 2 mg/mL VTE caused a decrease of 0.07 after 28 days and 8 mg/mL of 0.395 after 28 days. Control, 2 and 4 mg/mL VTE conditions allowed maintenance of stable texture. The AOC increased during storage in a VTE dose‐dependent manner (Figure 5c). And the increasing values of the same VTE concentration before 14 days perhaps due to binding of milk protein by gradual release of phenolic compounds during storage (Du et al. 2021; Ghorbani Gorji et al. 2015).

Previous work has shown that a 5% green tea infusion elicited superior *Lactobacilli* viability in probiotic milk compared with 10% or 15% during 21 days of storage, with higher green tea concentrations producing higher pH values (Najgebauer‐Lejko 2014). Thus, green tea extract had an effect on fermented milk that was proportional to its concentration, consistent with the current findings. In summary, 2 mg/mL VTE achieved optimal *L. casei* viability and pH during cold storage of fermented RSM.

## 4 Conclusions

The aim of the current study was to assess the influence of vine tea extract on *L. casei* fermentation of RSM. 2 mg/mL VTE stimulated *L. casei* growth and promoted RSM acidification. High (8 mg/mL) concentrations of vine tea extract reduced *L. casei* cell viability and maintained a higher pH, perhaps due to the pronounced antibacterial activity of DMY, the principal flavonoid compound in vine tea (Luo et al. 2023). DMY was extracted at over 50% efficiency. High DMY concentrations led to increasing total flavonoid content and AOC with increasing VTE concentration. Increased VTE concentration aided the maintenance of AOC during cold storage, extending shelf life. 2 mg/mL VTE maintained highly viable *L. casei* and stable pH during storage.

In summary, an appropriate addition of vine tea extract may be suitable to increase the flavonoid content and probiotic activity of dairy‐based health products.

## Author Contributions

**Chengjie Ma:** conceptualization (lead); data curation (equal); formal analysis (equal); funding acquisition (lead); investigation (equal); methodology (lead); project administration (lead); resources (lead); software (equal); supervision (lead); validation (lead); visualization (equal); writing – original draft (equal); writing – review and editing (lead). **Kun Wang:** data curation (equal); formal analysis (equal); software (equal); validation (equal); visualization (equal); writing – original draft (equal). **Man Zhang:** software (equal); writing – original draft (equal).

## Ethics Statement

The authors have nothing to report.

## Conflicts of Interest

The authors declare no conflicts of interest.

## Supporting information


Data S1.

## Data Availability

Data available on request from the authors.
