# Supplementary material for: Vine Tea Extract Enhanced the Fermentation of Skimmed Milk by Lacticaseibacillus casei
Source: Food Sci Nutr. 2024 Oct 22;12(11):9664–72. doi: 10.1002/fsn3.4547 (PMC11606875; doi:10.1002/fsn3.4547)
Supplement: Supplementary file 1 — Data S1. [file FSN3-12-9664-s001.docx]

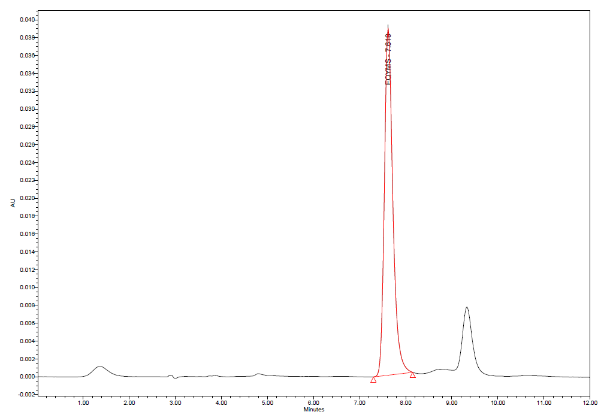

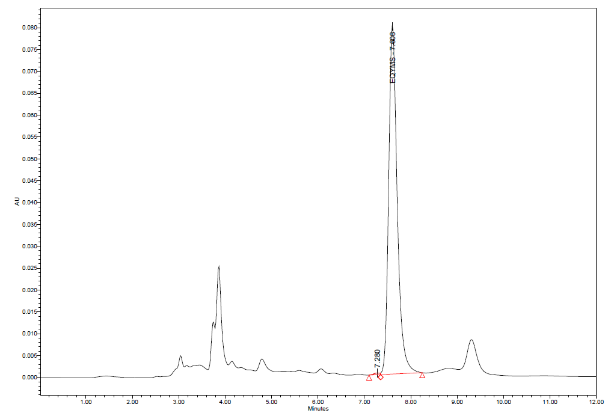


A

B

Fig. 1 DMY concentration in vine tea extract detected by HLPC

Note: The capital number A and B represented 50 and 100 μg/mL of vine tea extract solution.
